# Opposing and Operated Side Electroacupuncture Generates Similar Analgesic Effects on Pain after Knee Surgery

**DOI:** 10.1155/2021/6616886

**Published:** 2021-04-23

**Authors:** Hai Huang, Xiuling Song, Jiayou Wang, Man Xing, Bingxin Kang, Chenghao Ma, Wenbiao Li, Wenjun Han, Lianbo Xiao, Yuelai Chen

**Affiliations:** ^1^Shenzhen Hospital, Shanghai University of Traditional Chinese Medicine, 16 Xiantong Rd. Luohu District, Shenzhen, Guangdong 518004, China; ^2^Shanghai University of Traditional Chinese Medicine, 1200 Cailun Rd, Shanghai 201203, China; ^3^Guanghua Hospital, Shanghai University of Traditional Chinese Medicine, 540 Xinhua Rd, Shanghai 200052, China

## Abstract

The purpose of this study was to investigate whether opposing electroacupuncture (EA) could produce similar analgesic effects as operated side EA after knee surgery in rats. Sprague Dawley rats were randomly divided into the sham surgery group, and three surgery groups: opposing EA, operated side EA, and model. After surgery, compared with the sham surgery group, three kinds of pain behavior test methods (mechanical withdrawal threshold (MWT), cumulative pain score [CPS], and mechanical hypersensitivity of knee) were used to assess the pain behavior of the rats in the surgery groups. After knee surgery, the three surgery groups were intervened for three consecutive days: EA on the nonoperated side in the opposing EA group, EA on the operated side in the operated side EA group, and no intervention in the model group. It was shown that MWT was higher and CPS was lower in the two EA groups than in the model group on the first and second days after surgery. On the third day after surgery, MWT in the two EA groups was the highest among the 3 days, CPS was the lowest among the 3 days, and the number of nonvocalizations in rats also increased compared with the model group. Moreover, the MWT of the nonoperated side increased more in the opposing EA group than in the model and operated side EA groups. This indicated that both opposing EA and operated side EA methods can be used to relieve pain after knee joint surgery.

## 1. Introduction

Total knee arthroplasty (TKA) is a common orthopedic surgical procedure [1]. With the global aging trend, the number of TKA surgeries in most countries, including China [2], has increased annually. It has been estimated that by 2030, the primary TKA is projected to grow 85% to 1.26 million procedures in the United States [3]. Furthermore, it increases the medical and economic burden [4, 5]. Postoperative acute pain management is very important. First, it affects patients' satisfaction with TKA [6, 7], and second, it is a risk factor for readmission and chronic pain after primary TKA [8–10]. Currently, all of these analgesic methods have been developed in many disciplines, with multimode analgesia as the main mode.

In previous studies, the most commonly used animal experimental model for postoperative acute pain was the hyperalgesia induced by the plantar skin incision model, which is useful for evaluating the phenotype of postoperative pain [11]. However, the pain mechanism of this plantar skin incision model cannot describe various complicated and multifactorial aspects of postoperative pain management, and the surgical site cannot be directly and accurately located to the knee joint. Therefore, a suitable rat model after TKA is necessary. The Knee Surgery Model (KSM), which was first developed and explored by Dr. Buvanendran et al. [12], is easy to replicate and is an animal pain model performed after TKA, easy to replicate, suitable for evaluating pain and function, and helpful in determining the best method of analgesia after TKA.

Acupuncture has always been a good supplement and alternative therapy for pain [13]. However, there are various acupuncture methods of treating pain diseases clinically, such as electroacupuncture (EA) [14], transcutaneous electrical acupuncture stimulation [15], auricular acupuncture [16], heat-sensitive moxibustion [17], and acupoint catgut embedding [18]. Opposing needling is used to treat pain in traditional Chinese medicine [19, 20]. For unilateral TKA, the opposing needling method is more valuable in avoiding the risk of infection of acupuncture at the operated and adjacent area and increasing the patients' acceptance of acupuncture based on the overall characteristics of *qi* and blood flow in the whole body [21]. In this study, a rat model of knee surgery was established to compare the analgesic effects of opposing EA group and operated side EA group.

## 2. Materials and Methods

### 2.1. Animals and Grouping

Forty-nine healthy adult Sprague Dawley rats, weighing 220–260 g, of both sexes, were included. They were provided by Beijing Weitong Lihua Experimental Animal Technology Co., Ltd. and raised in a 12 h/12 h light/dark cycle environment with five rats in each cage in the laboratory animal center of Shanghai University of Traditional Chinese Medicine. They were quiet, at 24 ± 1°C and humidity of 60 ± 5%. The experiment was approved by the Animal Room Ethics Committee of Shanghai University of Traditional Chinese Medicine (No. PZSHUTCM190830007). We strictly referred to and implemented the ethical guidelines of the International Association for the Study of Pain [22], to reduce the number of animals used and the pain suffered by animals in the process of experiment.

All Sprague Dawley rats were numbered according to their weight and then randomly divided into four groups as follows: the sham surgery group (12 rats), and three surgery groups (the opposing EA (12 rats), operated side EA [13 rats], and model [12 rats] groups).

### 2.2. Knee Surgery Model

**Figure 1:**
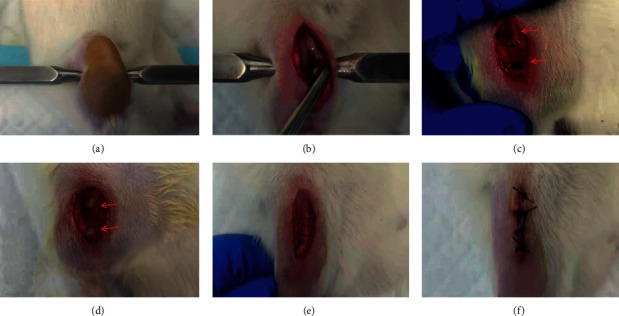
Photographs of the procedure of KSM.

This study used the knee surgery rat model developed by Dr. Buvanendran et al. We slowly poured 1.5% isoflurane into the air anesthesia machine, the pipe switch was adjusted to ensure that the induction concentration of anesthesia was 4%, and the concentration was maintained at 2%. From the supine position, the rats were bent 90 degrees after depilation around the right knee joint and firmly supported on both sides by the blunt end of the stereotactic ear rod (Figure 1(a)). The right knee joint of the rats was wiped with iodophor and then disinfected with 75% ethanol to remove iodine, and a sterile hole towel was spread. A 1 cm long skin incision was made on the patellar tendon with a #11 scalpel. The fascia on the muscles and tendons was scraped off with an elevator. The lateral tendon and inferior fascia were freed with the tip of a #15 scalpel blade; then, the tendon was moved laterally by approximately 3 mm and fixed in place with a retractor (Figure 1(b)). Using a high-speed drilling machine, a hole with a diameter of 1.4 mm and a depth of 0.5 mm was drilled in the femur and tibia (2 mm above and below the knee joint, respectively) (marked two red arrows in the Figure 1(c)). The two holes were immediately filled with rapid hardening of the dental cement (marked two red arrows in the Figure 1(d)). After the dental cement solidified, the mouth was soaked with povidone iodine disinfectant for 1 min and then rinsed with 0.9% normal saline to confirm that there was no bleeding point. The retractor was taken down, and the patellar tendon returned to the midline. A 5–0 nylon band needle suture line was used to suture the tissue incision of each layer (Figure 1(e)), the wound was wiped with a cotton ball and soaked in antibiotic solution, and the skin was finally sutured (Figure 1(f)). The rats were anesthetized and resuscitated in a clean incubator at 35°C for 30 min to 1 h.

The anesthetic method remained the same; the sham surgery animals only had a skin incision on the knee.

After the recovering from anesthesia, the rats were placed in the test cage to adapt for 20 min. Pain was measured, and the mechanical withdrawal threshold (MWT) was measured. The pain protective behavior appeared, and the decrease in pain threshold indicated that the model was successful.

### 2.3. Intervention

**Figure 2:**
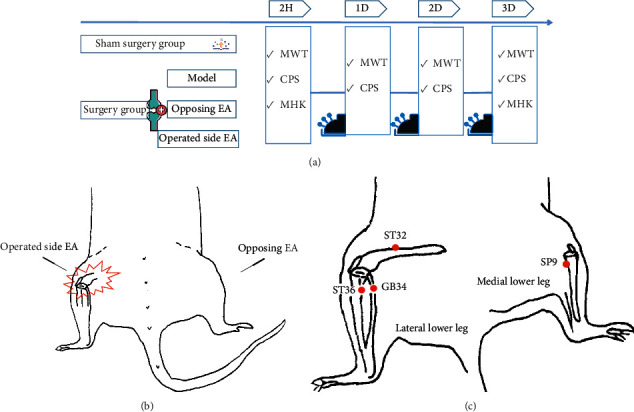
Experimental procedures, acupoints, and intervention. (a) Abbreviations: MWT, mechanical withdrawal threshold; CPS, cumulative pain score; MHK, mechanical hypersensitivity of knee. (b) SD rats received KSM on the right knee joint, EA on the the contralateral (left) side in opposing EA group, while EA on the operated (right) side in the operated side EA group. (c) The acupoints futu (ST32), housanli (ST36), and yangling quan (GB34) are located on lateral lower leg, yingling quan (SP9) on the medial lower leg, all are around the knee joint of rats.

Rats were intervened continuously for 3 days, once a day, for 30 min in each intervention (Figure 2(a)).

#### 2.3.1. Opposing EA Group

On the first day after surgery, the rats were bound and fixed on the acupuncture fixator, and then aseptic acupuncture (0.25 mm in diameter, 13 mm in length, Hwato Brand, Suzhou Medical Appliance Factory, China) was inserted to four points of the nonoperated side of the rats (Figure 2(b)), namely, Futu (ST32), Housanli (ST36), Yanglingquan (GB34), and Yinlingquan (SP9) (Figure 2(c)) [23, 24], with a 2 mm depth. The two connecting wires of the EA instrument were, respectively, connected at two pairs of acupoint needle handles (GB34 to ST32, ST36 to SP9), with a continuous wave of 2 Hz and a strength of 1 mA.

#### 2.3.2. Operated Side EA Group

On the first day after surgery, the rats were bound and fixed on the acupuncture fixator, and then aseptic acupuncture (0.25 mm in diameter, 13 mm in length, Hwato Brand, Suzhou Medical Appliance Factory, China) was inserted to four points of the operated side of the rats, namely, Futu (ST32), Housanli (ST36), Yanglingquan (GB34), and yinlingquan (SP9), with a 2 mm depth. The two connecting wires of the EA instrument were, respectively, connected at two pairs of acupoint needle handles (GB34 to ST32, ST36 to SP9), with a continuous wave of 2 Hz and a strength of 1 mA.

#### 2.3.3. Model Group

On the first day after surgery, the rats were bound and fixed on the acupuncture fixator without intervention.

#### 2.3.4. Sham Surgery Group

On the first day after surgery, the rats were bound and fixed on the acupuncture fixator without intervention.

### 2.4. Pain Measurements

#### 2.4.1. Mechanical Withdrawal Threshold Measurement

The MWT of 50% was calculated by the “up-and-down” method with von Frey hair [25]. Six von Frey hairs (6 g, 8 g, 10 g, 15 g, 26 g, and 60 g) were selected to detect the threshold of 50% mechanical stimulation foot contraction in rats on the operated side and nonoperated sides. To avoid the deviation caused by the rat's inadaptation to the test environment, the rat was placed in the pain test bracket (with 12 separate transparent plastic cages of 20 cm × 20 cm × 15 cm, and the bottom layer being a metal mesh of 1 cm × 1 cm) for 20 min. After the rats were used to the test bracket, firstly, from 8 g, the hairs were vertically pricked to the right foot center (operated side) of the rats through the mesh. The stimulation lasted for 5–8 s, and the contraction reaction of the rats was observed. The rapid foot withdrawal or licking reaction of the rats when stimulated, or the removal of the von Frey hair was marked as a positive reaction, represented by “X”; if there was no such reaction, it was regarded as negative reaction, indicated by “O”. If the foot contraction reaction was negative, the adjacent increasing von Frey hair stimulation was selected, whereas if the foot contraction reaction was positive, the adjacent decreasing von Frey hair stimulation was selected. The conditions for the end of the test were as follows: (1) it is still negative until the maximum stimulation intensity of von Frey hair is 60 g, and (2) after the first positive reaction, the up-and-down method was used four times for continuous measurement. After the test, the foot-shrinking reaction mode (g) was converted into the corresponding foot-shrinking threshold (N) using the following specific formula: 50% (g) threshold = (10^(*xf*+*kδ*)^)/10000, where *Xf* is the logarithmic value of the last test fiber wool; *K* is obtained by looking up the table and shrinking the foot reaction mode; and *δ* is the mean value of the logarithmic difference among six von Frey hairs. The plantar position of the nonoperated side to measure the mechanical pain threshold was the same as that of the operated side, and the test time was performed within 10 min after the MWT of the operated side was measured.

The test time was 2 h and 1–3 days after surgery.

### 2.5. Cumulative Pain Score

The cumulative pain scoring method of the model was as follows: the rats were placed in plastic cages on the pain test bracket, and the method of adapting to the test environment was the same as described in the abovementioned section. The position of the right hind paws of the rats was closely observed within 1 min and repeated every 5 min. The observation procedure lasted 1 h (12 recordings). According to the position of most right hind paws during this 1 min observation period, 0, 1, and 2 scores were given, where (1) score = 0: it can completely contact the grid floor, and it is completely load-bearing; (2) score = 1: the rear paws are partially or just in contact with the grid floor and are partially load-bearing; and (3) score = 2: the hind paws are completely separated from the grid floor or suspended on the tail and cannot bear the load [11].

The test time was 2 h and 1–3 days after surgery.

### 2.6. Mechanical Hypersensitivity of Knee

The hyperalgesia reaction was evaluated by squeezing the knee joint as follows: the knee joint was held with the thumb and forefinger and squeezed firmly (constant pressure kept for 3 s) to determine the hyperalgesia response of the rats. The hyperalgesia reaction during five trials was recorded, and each operation was recorded as Yes/No. The data were analyzed as the number of times that nonvocalizations were made [12].

The test time was 2 h and the 3rd day after surgery.

### 2.7. Statistical Analyses

The figures were drawn using GraphPad Prism 6 (GraphPad Software, San Diego, CA, USA). SPSS statistical software (SPSS version 26.0, IBM Corp, New York) was used for statistical analyses of the experimental data. Univariate analysis of variance (ANOVA) was used for the measurement data with normal distribution and homogeneity of variance among the groups. The Bonferroni method was used when the variance was homogeneous, whereas the Tamhane method was used when the variance was not uniform. The Kruskal-Wallis nonparametric test was used for non-normally distributed data. The pain behavior data collected belong to repeated measurement data and the normal distribution as means ± standard deviations ($\bar{x}\pm \text{s}$). Repeated measurement data ANOVA was used. If there was an interaction effect, the individual effect needed to be further analyzed. If it did not meet the conditions of repeated measurement ANOVA, a mixed model was established and the generalized estimation equation of repeated measurement data was used for statistical analyses. The difference between the data of each group was compared, and a *P* < 0.05 was considered statistically significant.

## 3. Results

### 3.1. General Condition of Animals

A total of 49 Sprague Dawley rats were purchased. Of those, 37 rats underwent surgery, of which 2 died due to excessive anesthesia. There were 12 rats in the sham surgery group, and 1 died because of excessive anesthesia. Therefore, this study effective study was 46 rats. After the surgery or sham surgery, the wound healed well, the fur was smooth and tidy, the appetite was good, and the activity was slow because of pain in the right lower limb. There were no significant differences in the weight of the rats before and after intervention among the four groups (*P* > 0.05).

### 3.2. Mechanical Withdrawal Threshold

#### 3.2.1. MWT of the Operated Side

**Figure 3:**
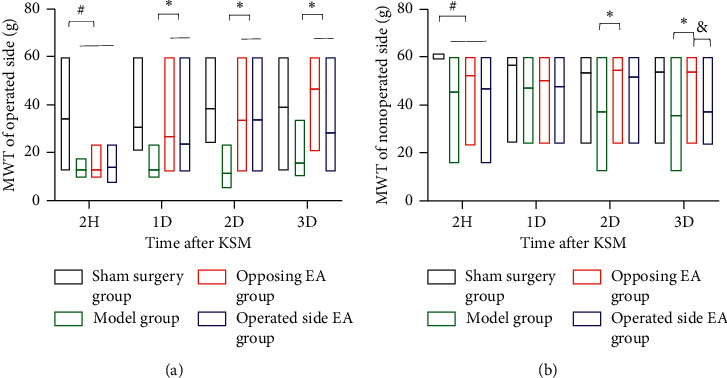
Mechanical withdrawal threshold. *Note.*^#^*P* < 0.05 compared with sham surgery group; ^*∗*^*P* < 0.05 compared with the model group; ^&^*P* < 0.05 compared with the operated side EA group.

As shown in Figure 3(a) and Table 1 and 2 h after the surgery, compared with the sham surgery group, the MWTs of the operated side among the surgery groups (including the model, opposing EA, and operated side EA groups) were decreased (*P* < 0.05), although there was no statistically significant difference among the three surgery groups (*P* > 0.05). From the first to third day after surgery, that is, from one to three cumulative interventions, the MWT of the two EA groups was higher than that of the model group (*P* < 0.05). There was no difference in the MWT between the first day (one intervention) and the second day (two cumulative interventions) after surgery in the opposing EA group (*P* > 0.05). There was no difference in the MWT between the second day and the third day (three cumulative interventions) after surgery (*P* > 0.05), although it was higher than that at the first day after surgery (*P* < 0.05). The MWT in the operated side EA group was improved, and there was no difference in the effect on the first, second, and third days after surgery (*P* > 0.05).

#### 3.2.2. MWT of the Nonoperated Side

As shown in Figure 3(b) and Table 1, 2 h after surgery, compared with the sham surgery group, the MWT of the nonoperated side among the surgery groups (including the model, opposing EA, and operated side EA groups) was decreased (*P* < 0.05), although there was no significant difference among the three groups (*P* > 0.05). Compared with the model group, the opposing EA group was beneficial to the improvement of the MWT on the nonoperated side and had this effect on the second and third days after surgery (*P* < 0.05), which was better than that of the operated side EA group on the third day (*P* < 0.05).

### 3.3. Cumulative Pain Score

**Figure 4:**
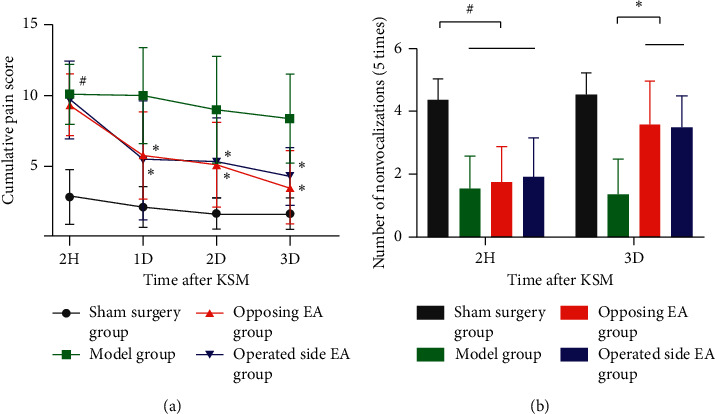
CPS and number of nonvocalizations. *Note.*^#^*P* < 0.05 compared with the sham surgery group; ^*∗*^*P* < 0.05 compared with the model group.

As shown in Figure 4(a) and Table 2, 2 h after surgery, compared with the sham surgery group, the cumulative pain score (CPS) among the surgery groups (including the model, opposing EA, and operated side EA groups) was increased (*P* < 0.05), although there was no significant difference among the three groups (*P* > 0.05). For three consecutive days after surgery, the CPSs in the opposing EA and operated side EA groups were lower than those in the model group (*P* < 0.05). Over time, there was no significant difference in CPS between the two EA groups (*P* > 0.05).

There was no significant difference in CPS on the first (one intervention) and second days (two cumulative interventions) (*P* > 0.05), although the score on the third day (three cumulative interventions) was lower than that on the first and second days after surgery (*P* < 0.05). In the operated side group, there was no significant difference in CPS between the first, second, and third days after surgery (*P* > 0.05).

### 3.4. Mechanical Hypersensitivity of Knee

As shown in Figure 4(b) and Table 2, 2 h after surgery, compared with the sham surgery group, the number of nonvocalizations (five times) among the surgery groups (including the model, opposing EA, and operated side EA groups) was decreased (*P* < 0.05), although there was no significant difference among the three groups (*P* > 0.05). On the third day after surgery, compared with the model group, the number of nonvocalizations in the opposing EA and operated side EA groups was increased (*P* < 0.05), and there was no significant difference between the two EA group (*P* > 0.05).

## 4. Discussion

As a kind of subjective discomfort, pain is often manifested in the behavioral representation of pain in animals, including lifting, withdrawing, limping, licking, and so on. In this study, two methods were used to calculate the MWT and CPS. During the study, the von Frey fair with corresponding g number was used to stimulate the same plantar position of the operated and the nonoperated sides. The rats' foot retraction reaction was observed, and the corresponding “X” and “O” marks were recorded. Finally, the 50% foot retraction threshold was calculated using the recorded data for statistical analyses. This method is widely used in the detection of mechanical pain threshold of animals [25, 26] and can well reflect the change in pain threshold of animals [27] and is a mature and recognized evaluation method for the measurement of pain behavior [28].

In the present study, it was found that both opposing EA and operated side EA could improve the mechanical pain threshold of the operated side of KSM rats, unlike those in the model group. There were a total of three interventions, and the effect was the best at the time of three cumulative interventions. The mechanical pain threshold of opposing EA group was similar to that of the operated side EA group.

Peripheral sensitization is characterized by (primary) hyperalgesia in the operated area, which in turn is characterized by a stronger response of sensitized nociceptors to suprathreshold stimuli and a lower activation threshold. Central sensitization is responsible for spreading pain and hyperalgesia to uninjured tissues (secondary hyperalgesia) and participates in the pain transmitting neurons of the central nervous system, usually located in the dorsal horn of the spinal cord [29]. With the development of central sensitization of pain, the threshold of mechanical pain on the operated side would decrease and the threshold of nonoperated side would also be decreased. From the statistical analyses, it was shown that opposing EA can improve the threshold of mechanical pain on the nonoperated side of rats. And, opposing EA was better than the operated side and model groups at the time with three cumulative interventions.

The CPS method was similar as the MWT test method. In this method, the behavioral performance of the lower limbs of rats with weight-bearing can be directly observed and recorded, and pain scores can be accumulated to evaluate the pain of rats after surgery or intervention. The method is simple and intuitive. In this experiment, the CPSs of the two EA groups were lower than those of the model group. The pain score of the two EA groups were the lowest at the time with three cumulative interventions, and then there was no significant difference between the two EA groups.

The method of knee mechanical hypersensitivity was referred to original paper of KSM [12]. For better presentation of the data and for easier comparison with activity monitor graphs, all the analyses and statistical data from the original literature were compared with the number of nonvocalizations (five times), regarded as the number of times that the rats did not experience mechanical hypersensitivity in their knee. Because the squeeze of the knee joint is a type of injury test, this experiment only evaluated two time points: one was to judge whether the model was successful or not within 2 h after surgery and the other was to judge the difference among the three groups after accumulating three interventions. It was found that the knee mechanical hypersensitivity reaction of the rats in the two EA groups was lower than that in the model group, and there was no significant difference between the two EA groups.

Through the measurement of the above three pain behaviors, it was verified that the KSM is also suitable for the study of acupuncture analgesia. The analgesic effect of the opposing needling method is equivalent to the analgesic effect of acupuncture on the operated side, which is similar to the results in many other research researches [19, 30].

We explored the regulation of pain after knee surgery in rats through opposing EA and operated side EA and found a similar analgesic effect of the two EA methods from the animal behavior characterization, which suggests the clinical application of opposing needling is an effective way of pain management after TKA surgery. However, the mechanism of acupuncture analgesia by opposing needling is unclear. Acupuncture signals are transmitted from the periphery, and the spinal cord is the first station to process pain and acupuncture information [31, 32]. After reaching the spinal cord, the “afferent impulse” caused by acupuncture mainly crosses to the contralateral ventrolateral tract of the spinal cord, which has a bilateral impact on the brainstem and the superior central nervous system [33, 34]. After the acupuncture information is transmitted to the supraspinal center, the analgesic mechanism is also involved in the regulation of the descending analgesia system, which originates from the brain stem and ends at the spinal cord [35, 36], and studies have shown the mechanisms of acupuncture analgesia may be involved in the alleviation of central sensitization [37]. Some functional magnetic resonance imaging studies have shown that contralateral acupuncture and ipsilateral acupuncture have different brain regulation mechanisms in which contralateral acupuncture regulates the anterior cingulate cortex, and other brain regions show analgesic effects [38, 39]. Some studies have also shown that contralateral acupuncture has a better effect on brain function than ipsilateral acupuncture [40]. Therefore, the limitation of our research is that we did not have a further trial for the analgesic mechanism of opposing needling.

## 5. Conclusion

Opposing EA can effectively improve the plantar mechanical pain threshold of operated side and contralateral side of rats after knee joint surgery, reduce the cumulative pain scores of rats, and reduce the mechanical hypersensitivity of the knee. Further, the analgesic effect is similar to that of the operated side EA method. This experiment provides an effective supplementary and alternative treatment for postoperative analgesia of TKA. However, the principle of its analgesic mechanism needs further study.

## Figures and Tables

**Table 1:** MWT of KSM rats (median (Q1, Q3), g).

	Group	*n*	2H	1D	2D	3D
Operated side	Sham surgery	11	29.35 (23.66, 54.59)	23.66 (23.66, 29.35)	34.07 (23.66, 60.00)	34.07 (23.66, 60.00)
	Model	11	12.39 (12.39, 14.21) ^#^	12.39 (12.39, 12.81)	12.39 (9.16, 12.39)	12.39 (11.30, 17.82)
	Opposing EA	12	12.39 (9.44, 13.47) ^#^	23.66 (17.77, 23.66) ^*∗*^	23.66 (17.87, 60.00) ^*∗*^	57.30 (23.66, 60.00) ^*∗*^
	Operated side EA	12	12.39 (9.79, 18.41) ^#^	23.66 (15.13, 23.66) ^*∗*^	28.87 (18.59, 58.65) ^*∗*^	23.66 (23.66, 28.79) ^*∗*^

Nonoperated side	Sham surgery	11	60.00 (60.00, 60.00)	60.00 (60.00, 60.00)	60.00 (60.00, 60.00)	60.00 (60.00, 60.00)
	Model	11	60.00 (22.98, 60.00) ^#^	60.00 (23.66, 60.00)	26.13 (12.39, 60.00)	23.66 (20.75, 60.00)
	Opposing EA	12	60.00 (40.55, 60.00) ^#^	60.00 (36.54, 60.00)	60.00 (55.94, 60.00) ^*∗*^	60.00 (60.00, 60.00) ^*∗*,&^
	Operated side EA	12	60.00 (23.15, 60.00) ^#^	60.00 (24.28, 60.00)	60.00 (40.55, 60.00)	26.51 (23.15, 60.00)

^#^ *P* < 0.05 compared with the sham surgery group; ^*∗*^*P* < 0.05 compared with the model group; ^&^*P* < 0.05 compared with the operated side EA group.

**Table 2:** CPS and number of nonvocalizations of KSM rats (mean ± SD).

	Group	*n*	2H	1D	2D	3D
CPS	Sham surgery	11	2.82 ± 1.92	2.09 ± 1.45	1.64 ± 1.12	1.55 ± 1.04
	Model	11	10.09 ± 2.12 ^#^	10.00 ± 3.41	9.00 ± 3.74	8.36 ± 3.14
	Opposing EA	12	9.33 ± 2.19 ^#^	5.75 ± 3.08 ^*∗*^	5.08 ± 3.00 ^*∗*^	3.50 ± 2.58 ^*∗*^
	Operated side EA	12	9.67 ± 2.74 ^#^	5.42 ± 4.21 ^*∗*^	5.25 ± 3.17 ^*∗*^	4.25 ± 2.05 ^*∗*^

Number of nonvocalizations	Sham surgery	11	4.36 ± 0.67	—	—	4.55 ± 0.69
	Model	11	1.55 ± 1.04 ^#^	—	—	1.36 ± 1.12
	Opposing EA	12	1.75 ± 1.14 ^#^	—	—	3.58 ± 1.38 ^*∗*^
	Operated side EA	12	1.92 ± 1.24 ^#^	—	—	3.50 ± 1.00 ^*∗*^

^#^ *P* < 0.05 compared with the sham surgery group; ^*∗*^*P* < 0.05 compared with the model group.

## Data Availability

The data used to support the findings of this study are available upon request from the corresponding author.
